# Efficacy of the Therapeutic Game “Trisquel” in the Treatment of Patients With Substance-Related Disorders Randomized Clinical Study

**DOI:** 10.3389/fpsyt.2022.864511

**Published:** 2022-05-02

**Authors:** Adolfo Piñón-Blanco, Esperanza Vergara-Moragues, Olga Gutiérrez-Martínez, Patricia Fernández-Palleiro, Sonia Rodrigues, Daniela Rodrigues-Amorím, María Teresa Lage-López, Ana González-López, Teresa Velasquez, Mónica Amorim, Manuel Lloves-Moratinos, Isabel Viéitez-Fernández, Gerardo Sabio-Fernandez, Rebeca Graña-Torralba, Vanesa Vilar-Díaz, Indalecio Carrera-Machado, Jesús Cancelo-Martinez, Adelino Ferreira, Susana Cardoso, Tania Rivera-Baltanás, Francisco Otero-Lamas, José Manuel Olivares, Carlos Spuch

**Affiliations:** ^1^Drug Dependency Assistance Unit of Vigo City Council (CEDRO), Vigo, Spain; ^2^Translational Neuroscience Group, Galicia Sur Health Research Institute (IIS Galicia Sur), SERGAS-UVIGO, Vigo, Spain; ^3^Departamento de Psicobiología y Metodología en Ciencias del Comportamiento, Universidad Complutense de Madrid (UCM), Madrid, Spain; ^4^Department of Psychiatry, Hospital Álvaro Cunqueiro, SERGAS, Vigo, Spain; ^5^Division for the Intervention of Addictive Behaviors and Dependencies (DICAD) of the Regional Health Administration-North of Portugal, Porto, Portugal; ^6^Picower Institute for Learning and Memory, Massachusetts Institute of Technology, Cambridge, MA, United States; ^7^Citizens’ Association for the Fight Against Drugs (Asociación Ciudadana de Lucha Contra la Droga), A Coruña, Spain; ^8^Comunidade Terapêutica Clínica do Outeiro, Porto, Portugal; ^9^Citizens’ Association for the Fight Against Drugs (Asociación Ciudadana de Lucha Contra la Droga), ACLAD-Alborada, Vigo, Spain; ^10^Ferrol Association of Drug Addictions of Ferrol (ASFEDRO), Ferrol, Spain; ^11^CIBERSAM, Centro Investigación Biomédica en Red Salud Mental, Madrid, Spain

**Keywords:** substance-related disorders, cognitive impairment, neuropsychological rehabilitation, psychoeducation, Trisquel

## Abstract

Substance-related disorders (SRD) have been consistently associated with alterations both in cognitive and executive functions, which affect to patients’ quality of life. The main objective of this work was to test the beneficial cognitive effects on patients with SRD after the implementation of “Trisquel,” an intervention program in board game format. To check the effectiveness of Trisquel program, a group of people diagnosed with SRD was randomly assigned either to the experimental group or to the control group. The experimental group performed Trisquel structured sessions twice a week during 3 months, while the control group performed routinely conventional therapeutic activities with the same frequency and duration. Neuropsychological tests were done to both groups before and after the intervention. After the 3 months of intervention the experimental group showed the following statistically significant improvements for WAIS-III subtests: number key, symbol search, arithmetic, direct digits, inverse digits, total digits, letters-numbers in the processing speed index and in the working memory index. Regarding STROOP tests, statistically significant progress was observed in the phonetic fluency letter P, phonetic fluency letter M, phonetic fluency letter R subtests, word-reading and word-color subtests. The control group only obtained improvements for WAIS-III subtests of arithmetic, letters-numbers and in the working memory index. The results of this study confirm that “Trisquel” is an effective intervention program for people diagnosed with SRD, getting improvements in processing speed (psychomotor and reading), attentional subprocesses (focused and sustained) and executive functions (updating and inhibition).

## Introduction

The Diagnostic and Statistical Manual of Mental Disorders DSM-5 (1) defines substance-related disorders (SRD) as those health problems caused by acute or chronic use of psychoactive substances. SRD are characterized by the association of cognitive, behavioral and physiological symptoms that lead a person to seek and use a substance despite its negative consequences. To date, SRD remains one of the most prevalent chronic diseases and there is evidence that the cost of treating Drug Use Disorders is much lower than not treating drug dependence. Despite the advancement in the knowledge of SRD and its treatments, relapse and therapeutic failure continue to be frequent problems (2).

Scientific evidence shows that drugs consumption has been consistently associated with the presence of alterations in different neuropsychological processes, such as memory, attention or executive functions (2–4), and these neuropsychological alterations are present even after prolonged periods of abstinence (5).

In Spain, 70% of people with SRD present cognitive impairment (2), which is considered a common feature in this type of patients (6). Literature highlights the relevance of cognitive rehabilitation in SRD and there is a growing number of studies proposing and evaluating different therapeutic interventions aimed at improving the cognitive domains in treatment outcomes (2, 7–9). Experience shows that psychotherapeutic interventions, such as psychotherapy or relapse prevention are failing in the goal of improve cognitive functions in SRD patients (10).

By other hand, an efficacy study of a Mindfulness and Goal Management Program carried out in Spain, has found an improvement in SRD patients regarding executive functioning, processes of reflection and goal achievement in daily activities (11).

### Trisquel

Given the clinical and social need to implement, invigorate and improve therapeutic and neurorehabilitative interventions in SRD patients, it was decided to create a new intervention program, called “Trisquel,” designed following the reference framework of a “serious game.”

“Serious games” do not have entertainment or amusement as their primary goal as are designed to educate, train or change behavior while entertaining players (12). Them have been developed in different formats encompassing both interactive role-playing games and board games (8, 13–18). In just over a decade, there has been an increase in its use as a tool to improve specific skills or competencies in different areas such as outreach, education, training, human resource management and health (12, 19, 20). Different studies on their effectiveness demonstrated that this type of therapeutic games create dynamic and motivating learning environments (13, 14, 19, 21–24). It has been noted that serious games combine three important aspects that contribute to the effectiveness of this approach: repetition, feedback, and motivation (8).

Previous experiences with serious games as “Road to awareness,” “The Trivia of Awareness,” “Escalation of Awareness,” “Trivia Psychotica,” “Reaction,” and “The Train” (21, 25–27), are clear examples of how a game can be a cognitive stimulation and rehabilitation tool, invigorating interventions, encouraging participation, providing information (psychoeducation) and working on social skills, problem solving, and deficit awareness.

For the design of “Trisquel” (22, 23, 28), the principles of neuropsychological rehabilitation have been taken into account. A neuropsychological assessment was performed, operational objectives were established, tasks were hierarchized and a continuous feedback system was provided. Besides that, different individualized strategies and techniques (restitution and compensation) (29) and others of group character (social skills) were combined and diverse theoretical reference models were taken into account.

The first “Trisquel” version began to be used in March 2008 and was completed in 2009 (Intellectual Property Registry VG 6-09) with a total of 590 theoretical-practical tests. Due to the good reception and evolution of the program, in 2014 a working group was created to carry out an extensive revision of the theoretical-practical contents of the entire program. Redundant, obsolete tests or those that did not provide information were eliminated, and the theoretical-practical contents of the program were expanded (28). To date, Trisquel has been used in over 12 centers in Spain and Portugal.

Although “Trisquel” has been tested in other populations with positive effects, the present work is the first one studying Trisquel effects on cognitive performance in SRD patients.

## Materials and Methods

### Design

Multicenter, longitudinal, prospective, randomized controlled study with pre and post neuropsychological measures.

### Study Population

Participants were selected among those patients under treatment in assistance devices [day unit (UD) and therapeutic community (CT)] of the Galician Network of Care for Drug Addicts (A Coruña, Ferrol and Vigo, Spain) and of the Division for the Intervention of Addictive Behaviors and Dependencies (DICAD) of the Regional Health Administration-North of Portugal (Porto, Portugal). This project was conducted between the years 2018–2020.

Inclusion criteria were: (1) diagnosis of substance-related disorders and other addictions according to DSM-5, (2) having capacity to consent (competence), (3) reading the project information sheet and signing informed consent, (4) being of legal age, (5) being able to read and write.

Exclusion criteria were (1) illiteracy, (2) diagnosis of intellectual disability (IQ < 70), (3) moderate or severe neurological damage, (4) suffering from an acute psychiatric process, (5) inability to be evaluated, (6) having an abstinence of less than 15 days, (7) not having cognitive impairment according, the Montreal Cognitive Assessment (MOCA ≥ 26).

A total of 101 people were evaluated, all of them meeting the inclusion criteria and none of them meeting the exclusion criteria. The total 101 were invited to participate, although only 71 patients completed the study. 30 patients (29.70%) dropped out the treatment. From Trisquel group 9 patients (30%) abandoned (3 of them from CT and 6 UD). The reasons for leaving the unit were 17 dropouts, 10 expulsions (due to positive controls and disciplinary reasons) and 3 for health reasons.

### Measuring Instruments

A questionnaire to collect sociodemographic data and tables shows a selected battery of neuropsychological tests (Tables 1, 2).

**TABLE 1:** Tests selected to build a battery of neurological tests and cognitive domains assessed.

Cognitive domain	Test
**MOCA**	
Screening for mild cognitive impairment.	Montreal Cognitive Assessment (MOCA)
**WAIS-III**	
Psychomotor processing speed and visual-motor coordination	Number key
Mental arithmetic and working memory	Arithmetic
Focused, sustained attention and working memory	Digits
Visual perception, psychomotor processing speed	Symbol search
Working memory	Letters and numbers
Psychomotor processing speed	Processing speed index
Working memory	Working memory index
**Test de STROOP**	
Speed of reader processing	Words
Selective attention	Color
Cognitive inhibition	Word-color
**Trace test**	
Sustained attention, motor, and visuospatial visual search skills	Part A
Alternating attention and cognitive flexibility	Part B
	Verbal fluency test
Functioning of the frontal lobe	Phonemic fluency
Functioning of the temporal lobe	Semantic fluency

**TABLE 2:** Descriptive analysis of sociodemographic variables.

	Trisquel group *n* = 40	Control group *n* = 31	*p*
Age^a^	44.28 ± 9.51	42.81 ± 9.64	0.335^d^
**Gender^b^**			1.000^c^
Male	33 (82.5%)	25 (80.6%)	
Female	7 (17.5%)	6 (19.4%)	
Cognitive impairment (MOCA)^b^	21.0 ± 3.3	21.3 ± 3.4	0.940^d^
**Level of education^b^**			^e^
<6 years of education	4 (10%)	2 (6.5%)	
6–9 years of education	28 (70%)	21 (67.7%)	
10–12 years of education	8 (20%)	6 (19.4%)	
>12 years of education	0 (0%)	2 (6.5%)	
**Primary drug**			^e^
Heroin	12 (30%)	8 (25.8%)	
Cocaine	17 (42.5%)	9 (34.6%)	
Alcohol	9 (22.5%)	11 (35.5%)	
THC	2 (5%)	2 (6.5%)	
Sedatives and hypnotics	0 (0%)	1 (3%)	
Age of drug initiation^a^	20.6 ± 7.7	20.6 ± 10.12	0.702^d^
Personality disorders^b^	10 (62.5%)	6 (37.5%)	0.775^c^
VIH+^b^	6 (15.0%)	0 (0%)	0.032^c^
VHC+^b^	12 (70.6%)	5 (29.4%)	0.268^c^
**Unit**			
Day unit	23 (57.5%)	14 (45.16%)	0.345^d^
Therapeutic community	17 (42.5%)	17 (54.83%)	

*^a^Values expressed as mean ± standard deviation.*

*^b^Values expressed as frequencies and percentages.*

*^c^Fisher’s exact test.*

*^d^T-test for independent samples.*

*^e^Cannot be calculated because there are categories with too few patients.*

• Montreal Cognitive Assessment, MoCA (30): The Spanish version of the MoCA test (designed by Nasreddine) was used, with a maximum score of 30, being the cut-off points (suggested by the author), 25/26 for mild cognitive impairment and 17/18 for dementia. Normacog scales were applied (31).
• Scale for the Measurement of Adult and Adolescent Intelligence (WAIS III) (32): The number key and symbol search subtests were used to calculate the processing speed index. The letter-number, digit and arithmetic subtests allow to calculate the working memory index. The Spanish adaptation of the WAIS-III was used. Reliability coefficients (two halves) range between 0.77 and 0.96.
• STROOP test (33): It is an instrument that allows a very brief and simple evaluation reading processing speed, the ability to focus and redirect attention and the ability to resist interference. The reliability using the test-retest method is.89 for Stroop-P, 0.84 for Stroop-C and 0.73 for Stroop-PC. Neuronorm scales were used for young adult population in Spain (34).
• TMT Stroop Test (35): The test consists of two parts, A and B. Part A assesses sustained attention, processing speed, motor, and visuospatial visual search skills. Part B assesses alternating attention and cognitive flexibility. Reliability is between 0.86 and 0.94%. Neuronorma scales were used for young adult population in Spain (36).
• Verbal fluency tests (37): Phonemic Fluency test, a task of oral production of words before phonetic instructions, and the Semantic Fluency test (animals), a task of linguistic production that requires the implementation of the mechanisms of access to the lexicon. Neuronorm scales were used for the young adult population in Spain (38).

### Procedure

The professionals who participated in the project received specific training on Trisquel’s methodology, dynamics and theoretical and practical contents prior to the start of the program. To avoid experimental biases and work overload in the centers, two psychologists were hired for the project to carry out the pre and post evaluations and the sessions with Trisquel in the respective centers.

All patients from the participating centers were filtered to meet the inclusion and exclusion criteria. On this filtered census, a simple random sampling technique was applied, until the estimated sample size was reached (between 6 and 14 patients per device). The individuals were then assigned to the experimental and control groups using a simple random sampling technique. All participants were provided a sociodemographic data collection questionnaire and a battery of neuropsychological tests before, and 1 week after the last intervention session. All tests were administered in two 30-min evaluation sessions under similar conditions.

### Ethical Considerations and Personal Data Protection

This project was approved by the Autonomous Research Ethics Committee of Galicia (Registration Code: 2018/153). All patients were informed about the rehabilitation program before its start. Patients read and signed the informed consent, voluntarily accepted their participation in the study and did not receive any financial or other incentive. The processing, communication and transfer of data was performed in accordance with the provisions of the European Data Protection Regulation (EU Regulation 2016-679 of the European Parliament and of the Council of 27 April 2016).

### Cognitive Rehabilitation

The experimental group was treated with Trisquel program and the control group received cognitive rehabilitation in the context of a biopsychosocial treatment, with a holistic and integrative approach, usually in the Treatment Facilities of the Drug Dependence Assistance Network of Galicia (Spain) and DICAD (Portugal). None of the two groups participated in any other rehabilitation program similar to the one proposed in this study. In order to carry out this work in the DICAD (Portugal), Trisquel has been translated and adapted to Portuguese language.

### Program Description

Trisquel program combines psychoeducation and cognitive stimulation strategies. Following the theoretical framework of “serious games: the principles of neuropsychological rehabilitation were taken into account and different individual techniques were combined, such as restitution and compensation (29), and others of group character, such as social skills (19, 20). Likewise, different theoretical reference models were taken into account, such as the Therapeutic Milieu, learning from successes, the information processing model of Miller (39), the working memory model of Baddeley and Hitch (40), the model of retrieval processes of information from memory of Moscovitch (41) and the emotional processing model of Ekman (42).

**FIGURE 1:**
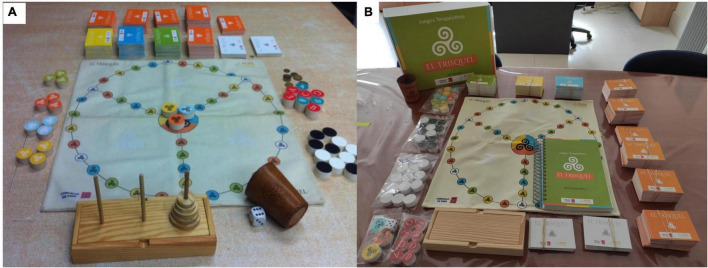
**(A)** Trisquel 2014 version, **(B)** box, professional’s manual and elements of the Trisquel 2014 game.

Trsiquel program consists on a board, cards and thematic blocks of cards with 1,105 theoretical-practical tests. The theoretical-practical questions and cognitive tests are organized in thematic blocks and were elaborated according to the specific intervention needs of the population to be treated (relapse prevention, social skills, HIV prevention, mental health, health interventions, sexually transmitted diseases, etc.) and to the cognitive characteristics of the patients (degree of difficulty, level of cognitive impairment). During the sessions, the difficulty of the tasks is graded by the moderator (member of the therapeutic team) according to the previous results obtained by the participants in the working memory index of the WAIS-III (digits, arithmetic and letters-numbers) and in the MOCA cognitive screening test. Although “Trisquel” includes other additional thematic blocks, for the purpose of this study were used relapse prevention and HIV-AIDS, women, drug addiction, gender violence, social skills (communication pragmatics), executive functions (emotional expression and recognition, planning, inhibition, mention theory), manipulative cognitive tests (psychomotor skills, visoconstructive praxis, instrumental skills) and non-manipulative tests (processing speed, attention, phonemic and semantic fluency, verbal and visual memory, mental arithmetic, topographical orientation), health interventions (sleep hygiene, nutrition), and smoking (information, prevention and treatment) (Figure 1).

Dynamics of the game could be compared to that of the popular trivia game. The main differences lie in the figure of the moderator, the graduation and hierarchy of the difficulty of the cognitive tests, the establishment of game rules that encourage participation and the content of the tests themselves (psychoeducational interventions related to the treatment of patients and cognitive stimulation tasks). Trisquel is a competitive game in which, at the end of the session, there is a winning team and the game ends when a test of each colored square (Green, Orange, Blue, Yellow) is correctly performed and the central square (starting place) is reached again. Trisquel sessions involve a maximum of 6 or 7 patients divided into three groups. The cognitive tasks are performed individually and the group tasks can be agreed upon and answered by the members of the group. Each session is structured in terms of working on theoretical and practical aspects. The blocks of cards are ordered numerically (from simple to complex) and by topics to be covered. Most of the program’s theoretical-practical tests can be the subject of intervention by the professional, providing clarifications on some theoretical or practical concept, behavioral modifications of the maladaptive behaviors that arise in the role-playing or positive modeling of such behaviors. Since its creation in 2008, Trisquel has gone through several versions and design improvements during previous pilot study processes. For a more detailed description of the program and its characteristics (23, 28).

#### Trisquel Group

**FIGURE 2:**
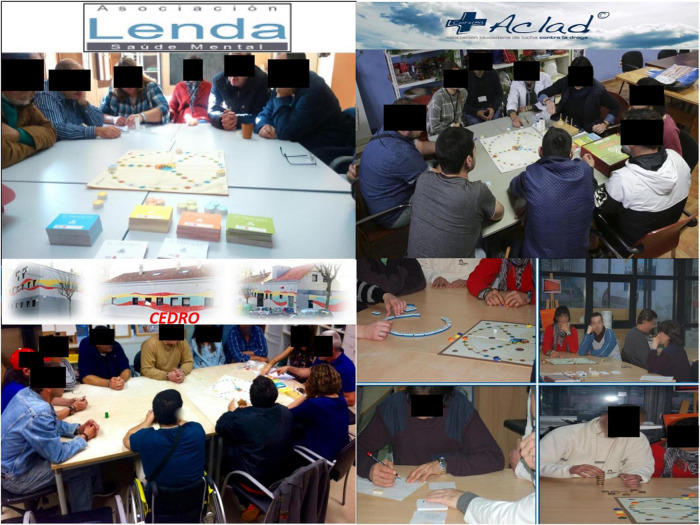
Pictures of Trisquel sessions in different rehabilitation centers.

The experimental group consisted on the administration of Trisquel program for 3 months, through 24 sessions of ± 60 min duration, with a frequency of 2 sessions per week, in the context of a usual biopsychosocial treatment in the participating centers. The sessions were carried out in groups of a maximum of 6 or 7 patients (Figure 2).

#### Control Group

This group carried out the routinely conventional therapeutic activities usually scheduled in each Care Center, consisting on Psychosocial Rehabilitation Support Programs (relapse prevention, occupational activities, and cognitive stimulation) implemented by a multidisciplinary team, with the same number of sessions (26), duration (± 60 min) and frequency (2 sessions per week) as the experimental group. The sessions were carried out in groups of a maximum of 6/7 patients. At the end of the study, all subjects in the control group were offered to perform the Trisquel program.

### Data Analysis

A descriptive analysis of the data was performed. Frequencies and percentages were calculated for qualitative variables. For quantitative variables, means and deviations were calculated. The normality of the variables in each of the study groups was tested using the Shapiro-Wilks test. To identify differences between the two groups (Experimental and Control), the Mann-Whitney *U*-test or the *t*-test for independent samples were used. For the pre-post intervention comparison, the Wilcoxon test or the *t*-test for related samples were used. Cohen’s D was also calculated to quantify the effect size. The programs used were SPSS v.19 and G-power.

## Results

Trisquel group consisted of 40 persons with a mean age of 44.28 years, of whom 33 (82.5%) were men and 7 (17.5%) women. Their mean MOCA cognitive impairment was 21.0 ± 3.3. Control group consisted of 31 persons with a mean age of 42.81 years of which 25 (80.6%) were men and 6 (19.4%) women. Their mean MOCA cognitive impairment was 21.3 ± 3.4. The descriptive analysis of the sociodemographic variables of each group is showed in Table 2. Statistically significant differences were found between groups in the variable’s Cannabis Addiction Diagnosis and HIV-associated organic pathology (Table 2).

### Intergroup Differences

No statistically significant pre-intervention differences intergroup were found in relation to neuropsychological variables.

### Intragroup Differences After the Intervention: Neuropsychological Assessment Module

In the cognitive performance of Trisquel group, statistically significant intragroup differences were found pre-post intervention in the subtests of number key (*p* = 0.001) with a moderate effect size (*d* = 0.763), symbol search (*p* = 0.001) with a moderate effect size (*d* = 0.598), arithmetic (*p* = 0.001) with a moderate effect size (*d* = 0.659), direct digits (*p* = 0.001) with a moderate effect size (*d* = 0.653), inverse digits (*p* = 0.025) with a small effect size (*d* = 0.385), total digits (*p* = 0.001) with a moderate effect size (*d* = 0.657), letters-numbers (*p* = 0.0001) with a large effect size (*d* = 0, 803) with WAIS-III, on the processing speed index (*p* = 0.001) with a moderate effect size (*d* = 0.598) and on the working memory index (*p* = 0.001) with a large effect size (*d* = 0.911) with WAIS-III, on the phonetic fluency subtest letter P (*p* = 0.001) with a moderate effect size (*d* = 0.756), phonetic fluency letter M (*p* = 0.004) with a small effect size (*d* = 0.488), phonetic fluency letter R (*p* = 0.002) with a small effect size (*d* = 0.575), in the word reading subtests (*p* = 0.007) with a small effect size (*d* = 0.372) and word-color of the STROOP test (*p* = 0.001) with a moderate effect size (*d* = 0.631).

In the cognitive performance of the control group, statistically significant intragroup differences were found pre-post intervention in the arithmetic (*p* = 0.003) with a small effect size (*d* = 0.488) and letter-number (*p* = 0.001) subtests with a moderate effect size (*d* = 0.652) and in the working memory index with WAIS-III (*p* = 0.002) with a moderate effect size (*d* = 0.572). Table 3 shows the mean scores and standard deviations of each group before and after treatment with respect to cognitive performance (Table 3).

**TABLE 3:** Intragroup comparison with respect to cognitive performance.

	Trisquel group				Control group			
	Pre	Post	*p*	Cohen d	Pre	Post	*p*	Cohen d
WAIS-III*^a^*								
Key numbers	34.5 ± 21.2–44.5	41.0 ± 25.5–58.2	*** (0.001)	0.763	39.0 ± 29.7–60.0	42.5 ± 23.5–59.5	0.339	0.121
Arithmetic	7.5 ± 6.7–11.0	9.0 ± 8.0–11.0	*** (0.001)	0.659	8.5 ± 7.0–9.7	10.0 ± 7.2–12.0	0.003	0.488
Direct digits	8.0 ± 6.0–9.0	9.5 ± 7.0–10.2	*** (0.001)	0.653	8.0 ± 6.0–10.0	8.5 ± 7.2–10.0	0.102	0.268
Inverse digits	5.0 ± 3.7–6.0	5.5 ± 4.0–7.2	* (0.025)	0.385	4.0 ± 3.2–5.7	5.0 ± 4.0–6.0	0.444	0.284
Total digits	11.5 ± 10.0–15.0	14.5 ± 11.0–17.2	*** (0.001)	0.657	12.0 ± 10,0–16,0	13,0 ± 11,2–16,0	0.085	0.362
Search symbols	24.0 ± 15.0–30.0	28.0 ± 18.0–34.0	*** (0.001)	0.598	23.5 ± 21.0–29.25	25.5 ± 20.0–30.75	0.079	0.277
Letters and numbers	7.0 ± 6.0–8.0	8.0 ± 7.0–11.0	*** (0.001)	0.803	7.0 ± 5.0–8.0	9.0 ± 7.0–10.0	0.001	0.652
Working memory index	83.5 ± 74.5–94.0	97.0 ± 85.2–106.0	*** (0.001)	0.911	90.0 ± 80.0–96.0	90.0 ± 83.0–102.0	0.002	0.572
Processing speed index	84.0 ± 77.2–92.0	92.0 ± 84.0–101.5	*** (0.001)	0.596	87.0 ± 78.0–98.0	92.0 ± 82.5–99.5	0.085	*** (0.001)
STROOP*^a^*								
Words	85.5 ± 71.5–96.2	91.0 ± 74.0 102.2	** (0.007)	0.372	89.0 ± 67.0–98.0	90.0 ± 86.0–109.0	0.107	0.322
Colors	57.5 ± 49.2–67.5	63.0 ± 55.0–68.7	0.061	0.239	61.0 ± 55.0–71.0	63.0 ± 60.0–68.0	0.073	0.254
Word-color	33.0 ± 26.2–41.0	39.5 ± 30.2–46.0	* * * (0.001)	0.631	37.0 ± 27.0–44.0	38.0 ± 29.0–44.0	0.559	0.059
TMT*^a^*								
Part A*^b^*	36.5 ± 28.7–56.5	35.0 ± 24.5–52.5	0.179	0.201	33.0 ± 22.0–47.0	28.0 ± 22.0–44.5	0.057	0.340
Part B*^b^*	60.0 ± 47.0–102.5	58.0 ± 46.5–75.0	0.093	0.546	79.0 ± 59.0–97.0	72.0 ± 48.7–86.2	0.064	0.689
Verbal fluency^3^								
Phonemic -P	12.0 ± 8.0–14.0	13.5 ± 11.7–17.2	*** (0.001)	0.756	12.0 ± 9.0–16.0	13.0 ± 10.2–17.5	0.351	0.166
Phonemic –M	11.0 ± 7.7–14.0	12.0 ± 9.0–15.0	** (0.004)	0.488	10.0 ± 8.0–14.7	11.0 ± 8.0–15.0	0.150	0.293
Phonemic -R	9.0 ± 7.0–12.0	10.5 ± 8.0–14.0	** (0.002)	0.575	10.5 ± 7.2–13.7	11.0 ± 7.2–14.0	0.666	0.094
Semantic	17.0 ± 13.0–21.25	18.0 ± 14.0–23.0	0.057	0.289	17.5 ± 14.0–20.0	17.0 ± 11.2–21.0	0.972	0.031

*^a^Values expressed as median ± interquartile range;*

^b^These scores are inverse, i.e., a higher score implies worse performance; Significance levels: *p < 0,05; ^**^p < 0,01; ^***^p < 0,001.

## Discussion

The aim of this study was to analyze the effect of the board game intervention program “Trisquel” on cognitive performance in people with SRD. The former clinical experience with Trisquel of several professionals in different centers after 13 years of implementation was suggesting that Trisquel helps to reduce therapist-patient distances, facilitates group dynamics, stimulates cognitive functions, is a versatile and adaptable tool to the needs of each patient (level of impairment) and allows the development of motivation on the part of the patients. It was also observed that Trisquel favors the incentive to win, the challenge of reaching a goal and the sense of efficacy, the feedback of progress, the greater adhesion of the members of the same team.

Due to these preliminary observations and the interest aroused by the program in recent years, it was decided to initiate a process of evaluation of the effectiveness of this therapeutic game in different populations (mental health and addictions). This study shows that Trisquel leads to improvements in processing speed, attentional subprocesses and executive functions in patients with SRD diagnosis. Overall, the results of this work are congruent with other studies that support the use of serious games in the clinical approach of patients with different mental disorders and, more specifically, it provides evidence on the usefulness of an intervention program in board game format to induce improvements in cognitive-executive functioning in SRD patients.

In order to be able to discuss the results, an interpretation of the results was performed following a structure based on a process analysis (processing speed, attentional subprocesses and executive components). To perform this analysis, different theoretical reference models were taken into account such as the factorial structure of attention (43), the clinical model of attention and the factorial model of executive components (44). Table 4 shows a comparison of the cognitive performance of both groups, where Trisquel group obtained statistically significant improvements in most of the cognitive-executive processes evaluated in this study.

**TABLE 4:** Process-based intragroup comparison of cognitive performance.

Cognitive-executive domain		Test	Trisquel group	Control group
			*p*-value	*p*-value
Processing speed	Psychomotor processing speed	WAIS-III Number Key	*** 0.001	0.339

		WAIS-III search symbols	*** 0.001	0.079
		WAIS-III processing speed index	*** 0.001	0.085
	Reader processing speed	STROOP word reading	** 0.007	0.107
	Visuospatial processing speed	TMT part A	0.179	0.057
Attention	Focused and sustained	WAIS-III direct digits	*** 0.001	0.102
	Selective	STROOP colored film	0.061	0.073
Executive functions	Executive update component	WAIS-III inverse digits	* 0.025	0.444
		WAIS-III total digits	*** 0.001	0.085
		WAIS-III arithmetic	*** 0.001	** 0.003
		WAIS-III letters and numbers	*** 0.001	*** 0.001
		WAIS-III working memory index	*** 0.001	** 0.002
		Phonemic fluency letter P	*** 0.001	0.351
		Phonemic fluency letter M	** 0.004	0.150
		Phonemic fluency letter R	** 0.002	0.666
Executive functions	Executive component of inhibition	STROOP color film word-color	*** 0.001	0.559
Executive functions	Executive component of change	TMT Part B	0.093	0.064

The values expressed in significance levels are: *p ≤ 0.05; **p ≤ 0.01; ***p ≤ 0.001.

According factorial structure of attention it is understood that processing speed reflects a basic property of the system where attention is implemented, and considers that it is a modulating factor of attentional performance (43). Trisquel group obtained statistically significant improvements in two of the three types of processing speed tasks evaluated (psychomotor and reading). These results are congruent with those obtained by other rehabilitation programs in which a significant improvement in processing speed is obtained after an intervention program (45, 46). A meta-analysis study on the evaluation of the efficacy of Goal Management Training concludes that intervention programs demonstrated efficacy in different populations by improving functioning in all cognitive-executive domains except processing speed (47, 48).

Regarding the attentional subprocesses performance, following the Clinical Model of Attention (one of the most referenced attention models in the literature), the results of the present study show that in Trisquel group statistically significant improvements were found in the subprocesses of focused and sustained attention, results in line with what has been found with other studies after the completion of a specialized rehabilitation program (45, 49). On the other hand, in control group no statistically significant improvements were found in any of the attentional subprocesses evaluated. These results may be consequence of the specific characteristics of Trisquel program (dynamic and motivating context, 266 cognitive-executive stimulation tasks, continuous reinforcement and feedback by the moderator, group dynamics, social exposure, etc.) or may be the result of the systematic repetition of a series of cognitive stimulation (restoration) exercises over a prolonged period of time. Overall, the results of this work are congruent with other studies that support the use of serious games in the clinical approach of patients with different mental disorders. Also, more specifically, this study provides evidence on the usefulness of an intervention program in board game format to induce improvements in cognitive-executive functioning in patients with SRD (13, 14, 19), in which it is consistently demonstrated that by challenging participants to think, explore and respond, they are motivated to learn new skills, knowledge, attitudes and behaviors, validating serious games as effective treatments of different psychiatric pathologies (21, 23, 24). Regarding executive functioning in the present study, and following one of the most referenced models of executive functioning in the literature (44), it was found that in the executive functioning of Trisquel group, statistically significant differences were found in the executive components of updating (working memory and phonemic fluency) and inhibition (cognitive).

In relation to the improvement of executive component of updating, the results of the present work are consistent with previous studies in which improvements in working memory are reported after the completion of a specialized rehabilitation program (2, 7, 11, 50, 51). Different studies show that the specific work of working memory is a facilitator of cognitive functions such as learning, verbal comprehension, thinking, reasoning or decision-making and a generator of an efficient buffering effect against attentional bias toward salient stimuli related to substance use (52, 53). Similar results to those obtained in the present study have been found in other populations with psychiatric pathologies (54, 55). Specifically, in a recent study assessing the efficacy of Trisquel in patients with a diagnosis of schizophrenia spectrum and other psychotic disorders, improvements in working memory were found after Trisquel program (23). Instead, other studies did not found benefits in specific working memory work, finding no effect on craving, substance use or attention bias (56). On the other hand, control group also obtained a statistically significant improvement in the executive updating component (working memory), indicative that biopsychosocial treatments with a holistic and integrative approach also generate changes in executive functioning, similar to those obtained with cognitive training programs. In this sense, this work may be one of the first studies that evaluates the efficacy of an intervention program in board game format compared to the holistic and integrative treatment model offered to patients with SRD in the care facilities (day unit, therapeutic community) of the Galician and Portuguese network of assistance to drug addicts.

In respect to the results obtained in the executive component of inhibition, Trisquel group obtained statistically significant improvements in the cognitive inhibition task (word-color) of the STROOP test, an executive component that allows us to stop an automated response and enables the inhibition of alternative learned behaviors as in this case represents the reading of words. These results are in line with other studies conducted with SRD patients, after the completion of a rehabilitation program such as Goal Management Training, one of the best validated interventions for executive dysfunction (47). Intervention program that has demonstrated its efficacy alone or in combination with other types of interventions such as mindfulness (11, 50). On the other hand, the control group obtained a worse performance in the cognitive inhibition task of the STROOP test.

Regarding the limitations of the present study, it can be said that the main one was not having studied the reasons for abandoning treatment, nor having correlated the different reasons for abandonment with the level of cognitive-executive deterioration of the patients studied. Another limitation was the absence of follow-up assessments. For this reason, no conclusions can be drawn as to whether the observed cognitive improvement contributed to maintaining abstinence in the following months, a fact found in other studies (57). In other studies it was found that the improvements observed at the end of cognitive training tend to be lost 6 months after the end of the intervention (45), an aspect that has not been assessed in this study either. Future efficacy studies should take these limitations into account.

## Conclusion

The creation of Trisquel in 2008 was motivated by the clinical need to dynamize the therapeutic interventions offered to patients. In this study, the aims were to dynamize the psychoeducational group (relapse prevention, social skills, HIV-AIDS) and to include neurorehabilitative interventions. For this reason, a game format was chosen, which allows to generate a therapeutic context that facilitates learning (therapeutic milieu) and allows to work *in vivo* on aspects of group interaction (social skills, emotional expression and recognition).

The present study has important implications for research and clinical practice. It provides explicit information for the planning of future studies (sample selection, assessment protocol, procedure, process-based analysis of results), and evidence of its feasibility. It represents an example of how an intervention program in board game format can generate a motivating therapeutic context that induces improvements in cognitive-executive performance, health perception, perceived symptomatology. It can be considered a versatile and dynamic tool that each professional can adapt to their daily intervention needs. This work shows how an intervention program in board game format integrated in a bio-psycho-social treatment with a holistic and integrative approach, can improve the cognitive-executive performance of patients with cognitive impairment under treatment in care facilities for people with substance-related disorders.

## Data Availability Statement

The raw data supporting the conclusions of this article will be made available by the authors, without undue reservation.

## Ethics Statement

The studies involving human participants were reviewed and approved by the Ethics Committee of Vigo, Pontevedra, and Ourense Registration Code: 2018/153. The patients/participants provided their written informed consent to participate in this study.

## Author Contributions

AP-B: conceptualization, methodology, writing—reviewing and editing, and supervision. EV-M: methodology and writing—original draft preparation. OG-M: methodology and investigation. PF-P, SR, ML-L, TV, MA, RG-T, AG-L, and VV-D: investigation. D-RA: writing—original draft preparation. ML-M, IV-F, GS-F, JC-M, AF, and SC: project administration. IC-M, FO-L, and JO: project administration, writing—original draft preparation, and supervision. TR-B: data curation and writing—original draft preparation. CS: data curation, supervision, writing–reviewing and editing, and funding acquisition. All authors contributed to the article and approved the submitted version.

## Conflict of Interest

The authors declare that the research was conducted in the absence of any commercial or financial relationships that could be construed as a potential conflict of interest.

## Publisher’s Note

All claims expressed in this article are solely those of the authors and do not necessarily represent those of their affiliated organizations, or those of the publisher, the editors and the reviewers. Any product that may be evaluated in this article, or claim that may be made by its manufacturer, is not guaranteed or endorsed by the publisher.
